# Modelling Multimodal Dialogues for Social Robots Using Communicative Acts

**DOI:** 10.3390/s20123440

**Published:** 2020-06-18

**Authors:** Enrique Fernández-Rodicio, Álvaro Castro-González, Fernando Alonso-Martín, Marcos Maroto-Gómez, Miguel Á. Salichs

**Affiliations:** Robotics Lab, Universidad Carlos III de Madrid, Av. de la Universidad 30, Leganés, 28911 Madrid, Spain; acgonzal@ing.uc3m.es (Á.C.-G.); famartin@ing.uc3m.es (F.A.-M.); marmarot@ing.uc3m.es (M.M.-G.); salichs@ing.uc3m.es (M.Á.S.)

**Keywords:** dialogue modelling, dialogue management, multimodal interaction, human–robot interaction

## Abstract

Social Robots need to communicate in a way that feels natural to humans if they are to effectively bond with the users and provide an engaging interaction. Inline with this natural, effective communication, robots need to perceive and manage multimodal information, both as input and output, and respond accordingly. Consequently, dialogue design is a key factor in creating an engaging multimodal interaction. These dialogues need to be flexible enough to adapt to unforeseen circumstances that arise during the conversation but should also be easy to create, so the development of new applications gets simpler. In this work, we present our approach to dialogue modelling based on basic atomic interaction units called Communicative Acts. They manage basic interactions considering who has the initiative (the robot or the user), and what is his/her intention. The two possible intentions are either ask for information or give information. In addition, because we focus on one-to-one interactions, the initiative can only be taken by the robot or the user. Communicative Acts can be parametrised and combined in a hierarchical manner to fulfil the needs of the robot’s applications, and they have been equipped with built-in functionalities that are in charge of low-level communication tasks. These tasks include communication error handling, turn-taking or user disengagement. This system has been integrated in Mini, a social robot that has been created to assist older adults with cognitive impairment. In a case of use, we demonstrate the operation of our system as well as its performance in real human–robot interactions.

## 1. Introduction

The field of social robotics has attracted a lot of interest in recent years and has begun to become a part of our daily lives. Several studies have aimed at using robots in tasks where human–robot interaction is involved, such as aiding people in need for 24/7 help or serving as companions [1,2,3]. These tasks require the elicitation of emotional bonds between robots and humans, similar to the one humans have with another living being [4]. For this to happen, robots need a set of skills that endows them with the functionalities required to interact with humans in a natural way [5,6].

In order to create an interaction that is perceived as natural by the human peer, roboticists need to take into account different factors. For example, in a human–human conversation, the speaker that is listening might take the initiative at any moment [7], forcing the other peer to adapt. In addition, information can be lost due to communication problems (e.g., a noisy environment). Timing is also a critical factor [8] during any dialogue because the response to an utterance can lose its meaning if it is not delivered in time. All of these situations are easily managed by humans but they become a challenge when we try to replace one of the speakers with a robot. Therefore, the development of a model that can represent a conversation while being able to adapt to any unforeseen situation has become a critical task in human–robot interaction (HRI) research.

When we talk about dialogue modelling, we refer to the formal characterisation of an interaction between two peers. This also implies maintaining a context that evolves during the conversation and managing the possible next steps that might be taken to progress on the dialogue [9]. In robotics, the module of the robot’s architecture that controls interactions between the robot and the users is called Dialogue System, or DS. It is in charge of tasks that range from handling inputs and outputs to controlling the flow of the interaction and managing errors [10]. Although the structure of a DS varies from one implementation to another, usually it has a Dialogue Manager (DM) at its core [11,12]. This module controls how the dialogue progresses and selects the appropriate output based on the inputs received and context information (past history, information from the environment, etc.). In a sense, dialogue modelling takes care of the theoretical aspects of designing a dialogue, while dialogue management represents a practical implementation of this theoretical model.

When modelling multimodal human–robot interactions, roboticists face issues that hinder the modelling process. Usually, they have to repeat the same structures for different scenarios and managing unexpected events results tricky. In addition, dialogues have to be fast enough to achieve the response times required for a natural interaction. Considering these issues, developing new robotic applications, where human–robot interaction is involved, is a difficult and time consuming task. Roboticists need tools that ease the interaction design process and allow them to focus on the logic of the interaction while they rid of the low-level, repetitive tasks.

In this work, we face the problems presented above with the design of a new approach for designing and managing multimodal human–robot dialogues. In our approach, any communicative interaction is modelled as a combination of four basic interaction units, called Communicative Acts, or CAs (we will refer to them as basic CAs). These atomic units, first presented in [13], control parts of the interaction and can be parametrised to adapt the interaction to different situations. The combination of basic CAs, in turn, results in more complex, re-usable blocks, which we called Complex Communicative Acts (CCAs), that, likewise, can be parametrised to adapt its functionality to different interactions.

The management of the interactions is divided in two levels: (i) the application level, where each robot’s application, using task-related information, decides how to combine and parametrise the required CAs (For the sake of simplicity, from now on, when using the term Communicative Acts, or CAs, we refer to both basic and complex Communicative Acts.); and (ii) the interaction level, where these CAs control the exchange of information with the user. They work with interaction-related information (e.g., what the robot has to convey to the user, or what information has to be provided by the other speaker) but without knowledge about the task that the robot is performing. Additionally, CAs provide several built-in recovery mechanisms to handle frequent problems that can arise during any dialogue (e.g., not obtaining a response from the other peer, communication errors due to perception problems and unexpected changes of topic during the conversation). This approach will ease the modelling of human–robot interactions by freeing up time from low-level interaction task development and thus allowing to focus on the combination and parametrisation of the CAs and CCAs.

Our social robots are designed for healthcare applications where the robot interacts with older adults that have mild levels of cognitive impairment. The robots serve as personal companions and also as an aid to the caregiver. In this context, we consider applications where a single patient, or a caregiver, interacts with the robot. With this in mind, when defining our approach to dialogue modelling, we focus on one-to-one interactions.

The main contributions of this work can be summarised as follows:

- The development of a two-level multimodal dialogue modelling strategy in which the applications of the robot control the flow of the dialogue using the approach that better suits their needs, and then model the actions taken in this interaction using atomic interaction units called Communicative Acts, or CAs. Each of these CAs is modelled as a state-machine-like structure that can be fully parametrised according to the needs of the interaction.
- The implementation of the HRI Manager, the dialogue manager in charge of managing the parametrisation and execution of the CAs, as well as solving certain communication problems that might arise during the interactions (user not responding, answer given by the user not matching the answer expected or malfunctions of the robot’s output interfaces, among others). The HRI Manager’s features include the assignment of priorities to each CA, parallel execution of CAs and built-in recovery mechanisms for communication errors.
- The development of a library of complex CAs, or CCAs that can be reused and parametrised in order to adapt them to different situations. These CCAs are combinations of basic CAs, and prove the modularity and re-usability of the approach presented.
- The integration of the HRI Manager in a dialogue system able to manage multimodal interactions with users in a real environment. In order to demonstrate the capabilities of our approach, we present a case of use, as well as performance statistics.

In the last part of the paper, we evaluate our system based on a case of use with four seniors that interact with our robot. Despite many researchers used subjective evaluation to test their dialogue systems, others conducted objective evaluations [10,14]. In this line, we decided to use an objective evaluation to demonstrate the capabilities of our HRI system for managing interactions appropriately. For this, first, we show how the CAs are applied in different interactions; second, we measure the response time of our system in order to conduct natural human–robot interactions; and, third, we show the utility of the built-in recovery mechanisms.

The rest of this manuscript is structured as follows. Section 2 gives an overview of the state of art in the field of dialogue modelling and analyses the the pros and cons of the each approach. Section 3 presents the theoretical framework behind the CAs. In Section 4, we present the robotic platform in which our system has been integrated, along with the technical aspects of the implementation of CAs. The repertoire of communicative acts currently developed can be seen in Section 5. Section 6 presents a case of use that demonstrates de operation of our system in real settings where our robot Mini interacts with seniors. In this section we also present the limitations of the work presented. Finally, Section 7 presents the conclusions extracted from this paper.

## 2. Related Work

Researchers have devoted great efforts to investigate dialogue modelling and dialogue management, from dialogue systems that use fixed representations of dialogues, to end-to-end systems that offer a high adaptability to multi-domain problems. In 2006, Trung proposed a categorisation of the different approaches to dialogue management [15], where he mentioned five categories: state-based, frame-based, plan-based, probabilistic-based and agent-based. Lately, a new approach has been introduced: end-to-end systems. In the following subsections, we introduce these approaches and present some of the most relevant contributions in each one.

### 2.1. State-Based Approaches

In *state-based DSs*, dialogues are modelled as finite-state machines. In 2010, Peltason and Wrede [16] developed a framework for developing mixed initiative interactions based on generic interaction patterns. These patterns represent coherent sequences of dialogue acts (for example, a question–answer sequence), and are implemented as statecharts, which are an extended form of finite state machines.

### 2.2. Frame-Based Approaches

*Frame-based DSs* define dialogues as a set of information slots that need to be filled during the interaction. One of the first frame-based DS was presented by Souvignier et al. [17] in 2000. In their paper they presented a set of technologies and strategies implemented in their spoken DS, including speech recognition, filtering and generation, natural language understanding and mixed-initiative dialogue management. The user is allowed to provide as much information as he/she wants and the system then uses this information to fulfil the user’s dialogue goal in the minimum number of dialogue turns.

In 2015, Alonso et al. proposed an augmented DS for social robots [10]. This DS uses information enrichment techniques for contextualising information provided by the user. This information is then used to fill information slots, changing the state of the dialogue and, therefore, advancing the interaction. More recently, Wessel et al. [18] proposed a platform named OntoVPA, an ontology-based DS for developing conversational virtual personal assistants. Their system provides ontologies for dialogue and domain representation that are used for analysing the user’s utterances and filling the slots. For dialogue management, an ontology-based set of rules tracks the state of the conversation and computes a response.

### 2.3. Plan-Based Approaches

*Plan-based DSs* see interactions as a mean for the speaker with the initiative to fulfil a goal or task with the collaboration of the other actors involved in the interaction. One of the first plan-based DSs was RavenClaw, by Bohus et al. [14]. RavenClaw is a plan-based DM that separates those skills that are domain-independent and those which need to be present in all of the dialogue, allowing the developers to focus only in modelling the control logic as a dialogue task tree. They aimed to facilitate the development of mixed-initiative DS.

### 2.4. Probabilistic-Based Approaches

In the last of Trung’s categories, *Probabilistic-based DSs* use probabilistic techniques to predict the state of the dialogue and the actions that have to be performed by the system. In 2015, Lison [9] presented a probabilistic-based DM that uses probabilistic rules (sets of rules that represent the transition and utility models of a dialogue POMDP). These rules are used to map logical conditions with probabilistic effects. This approach allows to reduce the amount of dialogue data required for parameter estimation. Based on the RavenClaw framework [14], Zhao [19] proposed a probabilistic-based dialogue management framework by formalising the dialogue task trees used by RavenClaw into Semi-Markov Decision Processes. Then, the system is trained using hierarchical reinforcement learning algorithms. This system also incorporates a domain-dependant ontology for encoding domain knowledge.

Recently, Milhorat et al. presented a dialogue manager for the android Erica [20]. This system consists of several modules that provide mixed-initiative dialogue based on information provided by the user and events detected by the robot’s sensors. A module in charge of answering questions and another one that generates statements provide the robot’s responses to any detected utterances. Probabilistic techniques are used to analyse the user’s utterance and generate a confidence score that is used to select between both modules. If no good response is found, then the DS provides modules that produce a backchannel fallback.

### 2.5. Agent-Based Approaches

*Agent-based DSs* are systems composed of several independent software agents that are in charge of different tasks during the interaction. The DM coordinates the actions of these agents. One example of agent-based DS is the work presented by Lee et al. [21], who proposed an example-based dialogue modelling approach for multi-domain DSs where the DM generates a structured query language statement based on the dialogue history and the current dialogue frame. The system then tries to match the current structure against a database of dialogue examples, which determines the next step that the system needs to take. This approach allows for a reduced dialogue corpus, simplifying the modification of dialogue policies in runtime. In this system, each agent manages a specific domain, and a generic dialogue frame provides generic functionality common to all dialogues.

### 2.6. End-to-End Approaches

The traditional approaches to dialogue management have the disadvantage of being too domain-dependant because they can only react to those user actions that match a closed set of structures (depending on the approach, these structures can be state-machines, information slots, sets of probabilistic rules, etc.) that have been previously manually encoded. The advances on Artificial Intelligence research has opened a new path in the field of dialogue management: end-to-end systems. In these systems, the managers try to overcome the domain constraint by learning from a corpus of previous dialogues without a need for assumptions about the domain. This reduces the cost of developing and maintaining multi-task DSs.

In 2016, Su et al. [22] proposed a two-step approach for dialogue management in task-oriented spoken DSs. During the first step, the system uses supervised learning and a dialogue corpus to train a neural network. The second step uses policy-gradient based reinforcement learning to improve the performance of the system. The same year, Cuayáhuitl et al. [23] presented a method for multi-domain dialogue policy learning based on deep q-networks. Their proposed method has two stages. First, they perform a multi-policy learning using a network of deep reinforcement learning agents. Each of these agents represents a specific skill for conversing in a particular sub-dialogue, and transitions among all agents are allowed. The second stage consists on compressing the raw inputs to create more compact state representations, which will be used to train the dialogue policies.

In 2016, Wen et al. [24] presented a neural network-based text-in, text-out dialogue system and a dialogue data collection method based on a Wizard-of-Oz framework. This modular end-to-end system uses a Long Short-Term Memory neural network (or LSTM network), called the intention network, to generate a distributed vector representation from a sequence of keywords. A series of belief trackers maps a sequence of sentences into a fixed set of slot-value pairs. Information in a database can also be accessed to answer certain petitions of the user (for example, if the user wants to know a place to eat, the system can search in the database for the information about restaurants). Using the set of slot values and the information from the database, a policy network generates a vector that represents the next action of the system. Finally, a response generation network uses this vector to generate the actual response of the system.

In 2017, Liu et al. [25] proposed a trainable neural network model for task-oriented DSs. They model task-oriented dialogue as a multi-task sequence learning problem that includes encoding user input, tracking belief state, issuing API calls, processing knowledge base results and generating system responses. An LSTM network is used to encode the sequence of turns in the dialogue. The final response of the system is generated from a skeletal sentence structure, a probability distribution of values for each slot in belief tracker and the information in the knowledge base that matches the user’s query. That same year, Li et al. [26] presented a learning framework for task-completion DSs, which is a variation of a task-oriented DS that aims to solve some problems of reinforcement learning-based approaches, such as only allowing one type of questions (a system that only understands yes/no questions, for example), poor robustness or handling user requests during dialogues. A language understanding module creates a semantic frame, classifies the domain of each user query and extracts domain-specific intents. The DM receives this information and performs a two stage process: dialogue state tracking and policy learning. The system actions are learnt from implicit dialogues, instead of incorporating state tracking labels. Supervised learning is used for starting the system, and then reinforcement learning is used to train it end-to-end.

In addition, in 2017, Eric et al. [27] proposed a neural dialogue agent for sustaining grounded, multi-domain discourse through a key-value retrieval mechanism. Instead of explicitly modelling dialogues through belief and user intent trackers, they use learned neural representations for implicit modelling of dialogue state. This key-value retrieval network is able to incorporate information from underlying knowledge bases. More recently, Budizanowski et al. [28] presented a multi-domain dialogue architecture with cross-domain database. They frame the dialogue as a context-to-response mapping and use an oracle belief tracker and a discrete database accessing component to augment a sequence-to-sequence model. In 2019, Lee et al. [29] presented ConvLab, an open-source multi-domain dialogue system platform designed for allowing researchers to set up experiments in the field of dialogue management. This platform offers annotated datasets and pre-trained models that can been used in different setups in order to compare approaches. It also provides end-to-end evaluation, both with real users and with simulators.

### 2.7. Comparison among Approaches

Considering that our field of application is HRI and the issues roboticists have to face when designing these interactions, we have analysed the approaches presented in this section according to three characteristics (this analysis is shown in Table 1):

- **Multimodality**: It refers to the use of multiple forms of expression (e.g., spoken language, gestures or touch) when communicating, both as inputs to and outputs from the dialogue systems.
- **Scalability**: This feature concerns how easy is to expand the system with new domains and tasks. This is related with the re-usability of already existing parts in new interactions.
- **Expressiveness**: It refers to the capability of the dialogues to generate and adapt their expressive responses to different circumstances of the interaction, such as different users or the time of the day.

In human–robot interaction, as well as in human–human interaction, multimodality is crucial. From the related works, just three of them (Pealtson et al. [16], Alonso et al. [10] and Lison et al. [9]) consider the use of different communication channels. The rest of the works are designed for verbal communication exclusively.

Regarding scalability and re-usability, there are certain similarities between approaches within the same DS category. For example, all end-to-end approaches can be adapted to new domains by generating new domain-specific training datasets, although the size of these datasets varies between the different models proposed. In addition, multi-domain end-to-end approaches that rely on the use of different agents for managing each domain would require also the development of new agents for the new domains.

When we talk about re-usability, end-to-end systems usually maintain the same model for all domains, so they provide a high re-usability. Frame based approach also tend to provide a similar scalability and re-usability factor. In order to adapt this type of DS to new tasks, the main requirement is to describe the task-specific aspects of the slot-filling process (number and type of slots, system responses, etc.), while they allow to re-use the slot-filling mechanism for all tasks. In this category, the approach proposed by Wessel et al. in [18] relies on ontologies, so in this case, adapt the system to new tasks, or domains, would require to expand the ontologies with domain-specific information. Pealtson et al. were able to overcome the general lack of scalability and re-usability of state-based approaches by finding generic interaction patterns that can be used to develop different tasks by parametrising these patterns differently.

The highest disparity between approaches can be found among the probabilistic-based ones, as the scalability and re-usability depend on how each system was designed. We can establish a difference between the work presented by Bohus et al. and the other three approaches presented. The former separates the domain-specific aspects from the domain-independent ones. This simplifies the development of new tasks, and allows to reuse the methods developed for managing aspects of the dialogue that are common to all domains. The latter approaches rely on probability rules, ontologies and a database that connects the user’s inputs with the system’s response, respectively. In all cases, adding new domains require either expanding the rules, the ontologies or the database. Finally, the agent-based approach presented relied on examples for deciding the system response to an user’s request. This system can be adapted to new tasks by adding new agents, while all the example-based dialogue modelling architecture can be re-used in different domains.

When analysing the expressiveness of every system, again we can find similarities among the approaches within the same DS category. End-to-end approaches generate system responses through Natural Language Generation, either based on templates or on language models. Frame-based approaches are the less flexible in this aspect, as system responses are defined when describing the task. Again, the biggest disparity comes when comparing the probabilistic-based approaches, as this systems’ response generation goes from using predefined responses to generating them through NLG. In Pealtson’s state-based approach, system responses can be modified by changing the parameters that define the generic interaction patterns, which is again an improvement over the traditional state-based approach, that tend to be less flexible. Finally, the agent-based approach presented by Lee et al. relies on template-based NLG for generating the system response.

### 2.8. Our Approach

Most of the works previously mentioned are exclusively focused on verbal communication, while multimodal communication has received considerably less attention. Only three of the reviewed approaches use multimodal inputs and outputs, while the rest of the solutions are either speech-based or text-based exclusively. Multimodality is a key feature in human–robot interaction since it directs its attention to provide an experience similar to the interaction between two human peers, which implies the use of multimodal communication. Therefore, we put special interest in developing a multimodal DS and on the extension of these approaches to a multimodal domain. Allowing multimodal communication will help to foster more natural interactions with humans.

Regarding flexibility, and scalability, our system can be compared with the solutions presented by Peltason and Wrede [16], as we look for common interaction patterns that can be parametrised to be applied to different interactions. Similarly to Bohus and Rudnicky’s work [14], we encapsulate those aspects of dialogue management that are domain-independent to simplify the development of new applications. The main difference with these works is two-fold. First, our CAs, which can be compared with Peltason and Wrede’s interaction patterns, include a series of built-in recovery methods for communication breakdowns and error handling strategies. Second, in our approach, we split the complexity of managing the interactions in two levels. In the application level, robot’s applications can focus on handling the interaction flow by combining and parametrising the CAs. Applications can use the dialogue modelling approach that better suits their needs for controlling the dialogue, as long as each step in the interaction can be modelled as a CA (using one of the existing ones or designing a new one). In the interaction level, these CAs take care of the low-level tasks of the communication with the users.

With the approach presented in this paper, our objective is to simplify the interaction design process and the addition of new robot’s functionalities. Roboticists that have to develop dialogues for new applications can focus on the high-level design of the interaction using the CAs straightforward, while they do not worry about the low-level tasks.

## 3. Modelling Human–Robot Dialogues

In linguistics, a speech act is defined as an utterance that does not have the goal to report something or describe a particular reality, but instead is meant to have an actual effect on the environment [30]. The idea is that utterances can be understood as actions, instead of a simple mean to convey a certain information. According to the theory proposed by J.L. Austin [30], these speech acts present three distinct levels:

- **Locutionary act**: The actual sentence being uttered, along with all aspects surrounding it.
- **Illocutionary act**: The intention that the speaker has when saying something; that is, what he/she wants to obtain with that utterance.
- **Perlocutionary act**: The action that is performed as an effect of the utterance spoken.

For example, if a person in a bar approaches a group of people sitting on a table and asks if any of the chairs they have is free, the locutionary act would be a question that aims to know if all the seats are occupied or not (“Is this chair taken?”). The illocutionary act would be the intention behind this question: in this case, taking one of the chairs. Finally, the perlocutionary act would be the effect that the question caused: either the speaker takes one of the chairs or he/she leaves because all are occupied. Among the three levels, the illocutionary act is the key one, because it represents the goal of the speech act. This has led to the term speech act referring sometimes to the illocutionary act, also known as Dialogue Act, or DA.

Dialogue Acts are categories that represent the intention behind utterances; that is, the conversational goal that the speaker wants to achieve, such as statements, clarifications, wh-questions or yes–no questions, among others. Being able to analyse utterances and classify them into one of the different DA categories is an important task when trying to develop systems that can automatically understand dialogues. We can also use these DAs as templates that will allow us to build new dialogues.

When analysing the commonly used DAs, we observed that they can be considered as particular cases of a more limited set of actions. For example, a wh-question and a yes–no question have different interaction goals, and so they are considered as different DAs. In the end, they both pursue a common goal: obtain a certain information from the other peer. Finding the actions that can be parametrised to obtain the DAs becomes the new objective. Our approach takes inspiration from these ideas and we propose to model all dialogues as a combination of basic interaction units that are in charge of handling the needed multimodal information. These actions are what we call Communicative Acts.

### 3.1. Communicative Acts

Communicative Acts [13], are the basic actions that will be used to model any human–robot interaction. The CAs are the atomic units that we can decompose a dialogue into, and can work either alone, or in combination with others. To find them, we decided to represent any human–human interaction as the product of two conversation’s dimensions: initiative and intention. Initiative represents which of the speakers is in control of the interaction. Usually, this means that he/she will be the one trying to advance the conversation while aiming to fulfil his/her communication goals. In the case of one-to-one interactions between a robot and a human, we have two possible situations: the robot is in control of the interaction, or the human is.

Intention defines the objective of the speaker with the initiative. After studying the different situations and needs that might arise during a dialogue, we came to the conclusion that all DAs have one of two goals: either the utterance aims to convey a certain information, without expecting anything back, or it aims to obtain information that the other speaker has. By combining both variables, we find our basic CAs: *Robot Asks for Information*, *Robot Gives Information*, *User Asks for Information* and *User Gives Information* (see Table 2).

The *Robot Gives Information* CA relays to the other peer a message using the appropriate modes of interaction, the *User Gives Information* CA controls all interactions where the user has the initiative but does not expect an answer from the robot, the *Robot Asks for Information* CA requests information from the user and waits for the response and the *User Asks for Information* CA manages user petitions when an answer is expected.

These CAs are designed to achieve these broad goals, which can then be configured to meet specific communicative objectives related to the application domain. For example, the *Robot Gives Information* CA, initiated by the robot and intended to provide some information, can be used by the robot to not only verbally greet a person but also to remind them about an event using a screen and the movements of the robot.

These four CAs are the fundamental building blocks that we use to model our human–robot dialogues. According to our modular approach, these pieces can be combined in parametrisable structures that are designed to be reused in different dialogues. We refer to these structures as Complex Communicative Acts (CCAs).

### 3.2. Complex Communicative Acts

Modelling all interactions as a combination of the four fundamental CAs simplifies the development of new dialogues because they provide the basic functionalities that are always required, such as managing errors that arise during a conversation. Consequently, developers only need to focus on how to combine these blocks to achieve different communication goals, applying the strategy for combining CAs that better suits each application.

However, although the CAs described in the previous section would be enough to model human–robot dialogues, this approach also has a drawback—if a certain combination of CAs appears several times in a single dialogue, then the developers would be forced to create this structure every time. To avoid this, we decided to create CCAs, which are predefined combinations of CAs that aim at achieving communicative sub-goals during human–robot dialogues. CCAs can be used as many times as needed in different dialogues, and they can be parametrised in order to fit the particularities of that scenario. Consequently, we try to simplify the process of dialogue creation, while letting the developers decide if they want to use these CCAs or use only the most basic elements.

The main characteristics of the CCAs are the reuse of combinations of basic CAs for achieving different goals and allowing an hierarchical dialogue design, where dialogues are a combination of CCAs that are also a combination of basic level CAs. It is important to mention that there is no limit for the number of CCAs that can be created because any dialogue structure can be modelled as one of these complex structures at the developer’s requirement. In this approach, it is crucial the identification of those combinations of CAs (i.e., CCAs) that appear multiple times.

## 4. The Robot Mini and Its HRI Architecture

**Figure 1:**
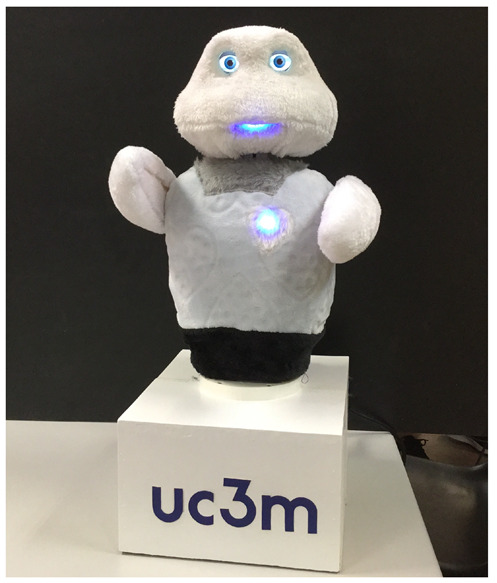
Mini, a social robot developed for interacting with older adults that suffer from mild cases of cognitive impairment.

The approach we have presented for modelling human–robot dialogues has been implemented in the social robot Mini. Mini, shown in Figure 1, is a social robot that is designed to interact with older adults with mild cases of cognitive impairment [31,32]. Is a tabletop robot with 5 degrees of freedom (two in the neck, one on each arm and the base), OLED screens in the place of eyes, a heart that can be lighted and a external tablet, for displaying multimedia content.

**Figure 2:**
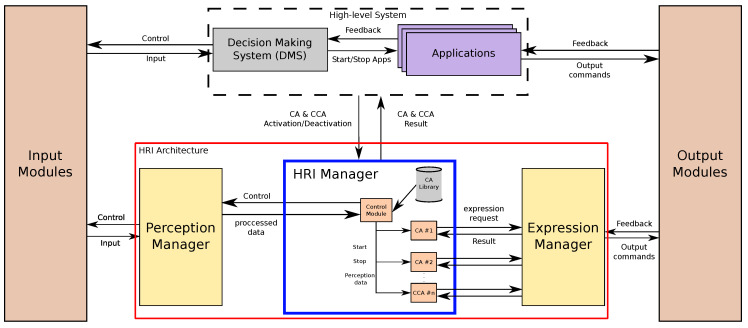
Black-box view of the human–robot interaction (HRI) Architecture (Red box) and its communications with the rest of the robot’s software architecture. This paper focuses on the central piece of the architecture, the HRI Manager (blue box).

The architecture of this robot works as shown in Figure 2. At the top, a Decision Making System (DMS) [33] controls all high level decisions and selects which of the robot’s applications has to be activated at any time. These applications provide all of the robot’s functionalities. For example, one application can be used to show different multimedia contents, such as videos or photos, while other can implement a game that the robot can play with the users. Based on the interaction with a user and other external aspects, the DMS decides when to activate or deactivate each application. On the other end of the architecture, a series of modules control all the input and output interfaces that Mini uses to retrieve information from the environment and for communicate information to the user. Between the robot’s interfaces and the top level of the architecture (the DMS and the applications), we can find the Human–Robot Interaction architecture, or HRI architecture (red box in Figure 2).

The HRI architecture is the part of the robot’s architecture that manages all interactions between the robot and the user. It receives all of the information coming from the input modules, packages it, advances the dialogue based on the information received and sends commands to the output modules to convey the required messages. At the core of this module is a key element in controlling interactions: our Dialogue Manager, which we have called HRI Manager (blue box in Figure 2). This module is in charge of processing the different CAs requested by the robot’s applications and configuring them to fulfil the application’s needs. In terms of HRI, the whole architecture works as a two-level system: (i) the applications have task-related knowledge and control the dialogue flow to achieve task-specific objectives by requesting the activation of different CAs; (ii) the HRI Manager uses interaction-related information to control the CAs, that are also in charge of handling errors that might arise during the interactions. This division of the dialogue management gives the roboticists the capability of building interactions that suit the needs of the particular applications, without having to micromanage all of the small tasks related to dialogue management.

The HRI Manager receives all of the input information from the Perception Manager, the module in charge of collecting all the perceptual information relevant for HRI. The different input interfaces send the perceived information to the Perception Manager, and this module filters, packages and sends it to the HRI Manager, that, in turn, distributes it to the active CAs that are expecting it. On the other end, the CAs communicate directly with the Expression Manager, which is the module that controls the output interfaces of the robot, when they are used for interaction purposes. When a CA needs to communicate a message, it requests this module the execution of the corresponding gesture with the right parameters. The Expression Manager receives these requests, schedules them and executes the different expressive actions that compose the gesture. The detailed operation of the Perception Manager and the Expression Manager are out of the scope of this work, we focus in the HRI Management.

### 4.1. Execution of CAs

Whenever one of the robot’s applications needs to interact with the user, the application requests the activation of the appropriate CA (basic or complex). This request specifies which of the CAs has to be executed, and the proper parameters (see Section 4.3). The HRI Manager will load the appropriate CAs, will configure them using the parameters contained in the activation request, and will execute them. If any of them needs information from the environment or the user, then the HRI Manager will configure also the input channels that will be used according to the parameters received. For example, if the *Robot asks for information* CA is loaded and the user’s answer has to come through a grammar-based speech recognition module, then the HRI Manager will request the speech recognition module to load the grammar required.

Each time the Perception Manager sends new input data related to HRI, the HRI Manager will relay this information to any active CA that is expecting it. The type of data that a CA receives can be configured. Thus, if a CA is configured to only receive information retrieved through the touch screen, the HRI Manager will not send the user’s utterance data to this CA. Once the CA completes its task, the HRI Manager relays the result to the application that requested the activation in the first place.

At any time, the applications can request the deactivation of any of the active CAs by sending a deactivation request with an ID associated to the CA to the HRI Manager. This is useful when a sudden change in the direction of the dialogue is required. When a deactivation request is received, the HRI Manager checks if the CA is active or not. If it is, it stops its execution. If it is not active but is waiting to be executed, then the HRI Manager just discards it.

### 4.2. Managing Multiple CAs

Our system needs to have multiple active CAs at the same time in order to, for example, handle a change of topic during an interaction. Thus, the HRI Manager needs to be able to manage multiple CAs concurrently. Parallel execution of CAs can be cumbersome because conflicts might arise if several of them need to use the same input communication channels at the same times. For example, if two CAs need to display a menu through the tablet, or use the same touch sensor, then assigning the input information to the correct CA becomes an almost impossible task. We decided to solve this problem by assigning priorities to each CA.

We defined three levels of priority: low, medium and high. The CAs of the task the robot is currently executing will have medium priority. For example, if our robot is playing a game with a user, then the application controlling the game will have medium priority. Critical tasks (e.g., reminding the user critical events, like taking his/her medicines) will be assigned a high priority and will be able to interrupt the current application. Finally, any task that provides not essential functionalities will have low priority. For instance, if the user asks the robot what time it is, then this is not considered critical so the robot will answer only if there is not a higher priority task running.

The priority system works different for CAs where the user has the initiative and for those where the robot has the initiative. We assume that there is no situation that would require two CAs where the robot has the initiative at the same time (e.g., it would make no sense to ask to the user two different questions at the same time, or ask a question and utter a statement that has no relation to the question). Based on this assumption, we decided that all CAs where the robot has the initiative would be executed sequentially, based on their priority.

When one of these CAs is requested, the HRI Manager assigns it to the corresponding queue depending on its priority level. If the new CA has a higher priority than the one currently active, then the manager will stop the current CA and store it at the beginning of the corresponding priority queue. Each time a CA ends, the HRI Manager executes the next one in order of priority (the priority queues follow a first-in, first-out approach).

In the case of CAs where the user has the initiative, we need to be able to maintain several of them active for the user to take the initiative and to control a wide range of different situations. When an application requests the activation of an user’s CA, the HRI Manager checks which output and input interfaces it requires. The new CA is only activated if the interfaces that it requires are not being used or can be shared among several CAs. For example, we cannot use the same touch sensor as an input for two CAs because there would be no way to know if the input should be assigned to the first or the second one, but we can maintain several CAs that expect an answer through voice because we can assign the utterance to the appropriate one based on the semantic values extracted from that utterance. If a conflict arises with any of the current CAs, then the one with the highest priority will remain active, while the other will be deactivated (if it was active) or discarded (if it is the new CA).

Finally, it is important to remark that any interaction where the robot has the initiative will always be prioritised over any of the user’s CAs if a conflict regarding the use of the interfaces arise. In this situation, the CA where the robot has the initiative will be executed and, once it is finished, the CA where the user has the initiative will be activated. We made this design decision because the CAs where the robot has the initiative are usually finite and will be executed immediately, while the CAs where the user has the initiative can be active indefinitely and might or might not be needed. We decided that it was preferable to not be able to respond to an user command for a short period of time (the time that it takes to execute the robot’s CA) than to skip one of the robot’s actions, which could affect the execution of the applications.

### 4.3. Parametrization of Basic and Complex CAs

When using the CAs, it is necessary to configure a series of parameters to adapt it to the specific needs of the application requesting its usage, and to the scenario in which the robot will be deployed. Each CA needs a unique ID that will be used to properly identify it, as several instances of the same CA can be used at the same time, and we need to be able to manage each of them individually.

In addition, the execution mode of the CA has to be configured. It can be configured either as *ending* or *continuous*. *Ending* CAs are those that are executed only once. After the CA finishes, the result of the interaction is returned (if it was a success, if it was cancelled, or if it failed due to an unexpected error), along with the user’s response, if necessary. This result is returned by every CA and CCA. Meanwhile, *continuous* CAs are active until a deactivation request is issued by the application that requested their activation in the first place. The result is returned when the execution is completed, just as it is for ending CAs, but this time the execution of the CA starts again.

All CAs can be configured either way but CAs where the robot has the initiative are usually configured as *ending*, while those CAs where the user is the one with the initiative are configured as *continuous*. This happens because while the robot applications can activate robot-initiated CAs at the exact moment we need them, we cannot foresee when the user will want to take the initiative, so we need to keep those CAs active and waiting for any information coming from the user. For example, during a quiz game where the robot is asking questions, the application would configure each of the questions as *ending* because they are delivered at specific times. At the same time, the user needs to be able to control the game and stop it at any moment, so the application would activate a CA to manage these commands. They can come at any time during the game, therefore the application would configure this CA as *continuous*.

As it was mentioned in the previous subsection, in order to deal with conflicts of different CAs using the same channel concurrently, a level of priority can also be assigned to each them. This priority can be high, medium or low, and it will be used to decide which CA gets to use the input and output communication channels in case several of them try to use them at the same time.

Each CA can be parametrised with the information needed to configure the different robot’s interfaces. In the case of CAs that need information from the user, the applications requesting their activation can define and configure the channel used to get that information. For instance, when using the information from the user’s utterance, CAs can be configured with the grammar that has to be loaded in an automatic speech recognition (ASR) module. When talking about CAs that use the expressive capabilities of the robot, applications can configure them to use multimodal expressions that have to be performed by the robot or even parametrize a particular output channel (e.g., change the robot’s gaze). This parametrisation allows us to adapt each generic CA and CCA to the particular needs of a specific task. The combination of properly parametrised CAs and CCAs will conform all of the dialogues that our robots require.

### 4.4. Handling Errors in Communication with Basic and Complex CAs

Any interaction is subject to uncertainties and errors during the communication that have to be managed to provide a natural interaction with the user. The modules in charge of processing user inputs and retrieving information from the environment can fail in their task due to internal problems or acoustic noise being present in the environment (e.g., the ASR module sometimes fails to identify the user speech if there is noise in the background, or if the user does not speak clearly enough, which would produce information with a low confidence). When this happens, the module in charge of processing input data sends a communication failure message, and the CA waiting for information coming through that interaction channel responds by asking the user to repeat whatever he/she has said or done. If the user was speaking to the robot, then there is another possible case: if the speech recognition module gives a result, but the confidence of that result is not high enough, then the robot will repeat to the user the word or sentence detected and them ask him/her to repeat the answer.

Another problem that might appear during an interaction involves the sudden loss of the other peer in the dialogue. Our system needs to manage this situation without getting stuck. To solve this problem, the *Robot Asking for Information* CA controls the time the user takes to answer to the robot’s question. If the user takes too much time, then the CA tries to encourage the user to answer and then repeats the question. If the same situation keeps happening, then the CA gives up and communicates the failure to the application that requested its activation. The time the CA waits before encouraging the user to answer can be adjusted according to the particularities of the user (for example, older adults might require more time to provide an answer than younger users).

## 5. Repertoire of Communicative Acts

In this section, we present the CAs, which serve as the fundamental building blocks that will conform all dialogues, as well as a series of complex structures that we have currently identified and modelled as CCAs based on the current application of the robot Mini (Section 4). It is important to notice that, while we consider that only four basic CAs exist, the repertoire of CCAs is not fixed and it grows as more of them are required and identified when designing the interaction.

### 5.1. Basic Communicative Acts

As mentioned earlier, the four fundamental CAs come from the combination of two dimensions of the human–robot communication: who is the peer with the initiative and what is the goal that this speaker wants to achieve with the utterance. Based on those two aspects, the resulting basic CAs are:

- **Robot Asks for Information**: The robot takes the initiative to obtain some information from the user. Both the request and the reply can use one or more communication channels (e.g., voice or touch). This CA can manage questions where the robot is looking for a single answer (e.g., how old are you?), or for a series of answers (e.g., tell me your daughters and sons’ names). In the latter, these answers might be requested in a specific order. The questions can then be closed, where the answers are limited, or open, where whatever the user answers is accepted (e.g., how was your day?).
- **Robot Gives Information**: This CA is used to convey certain information to the user, which will be displayed through the appropriate communication channels. For example, when the robot greets someone, it can use a *Robot Gives Information* CA, so it does not expect an answer. In this example, the robot can convey the message by saying “hello!” and waving an arm.
- **User Asks for Information**: This CA is used when the user asks something to the robot and expects an answer from the robot. This CA receives the user’s question through the perceptual capabilities of the robot, processes it and then, once the answer is ready, it communicates it to the user. For instance, a *User Ask for Information* CA can be used to respond with the user asks the robot for the current time.
- **User Gives Information**: This CA waits for the user to convey a specific information and sends it to the robot’s application/s that can process it. In this case, the user does not expect an answer from the robot. This type of CA is used when the user wakes up the robot by touching it.

In basic CAs, developers can parametrise the message that has to be conveyed through each of the output interfaces of the robot. For the CAs used for processing information given by the user, the input channel can be parametrised, along with an identifier for the answer (this is only used for identifying the grammar-based ASR responses), the time the user has for answering (only for the *Robot Asks for Information* CA), and configuration data for the input channels (specifically, the grammar that has to be loaded in the ASR module and the configuration of a menu that has to be displayed in the tablet, if needed).

### 5.2. Complex Communicative Acts

As mentioned earlier, CCAs are enclosed combinations of CAs that can be easily reused. Considering the robot Mini will perform cognitive stimulation exercises with older adults (more details are presented in Section 6), we have identified the following CCAs: *Right–Wrong Question CCA*, *Question with Confirmation CCA*, *Switching Mode Question CCA*, *Manage Multimedia Content CCA* and *Communication Warning CCA*.

#### 5.2.1. Right–Wrong Question CCA

**Figure 3:**
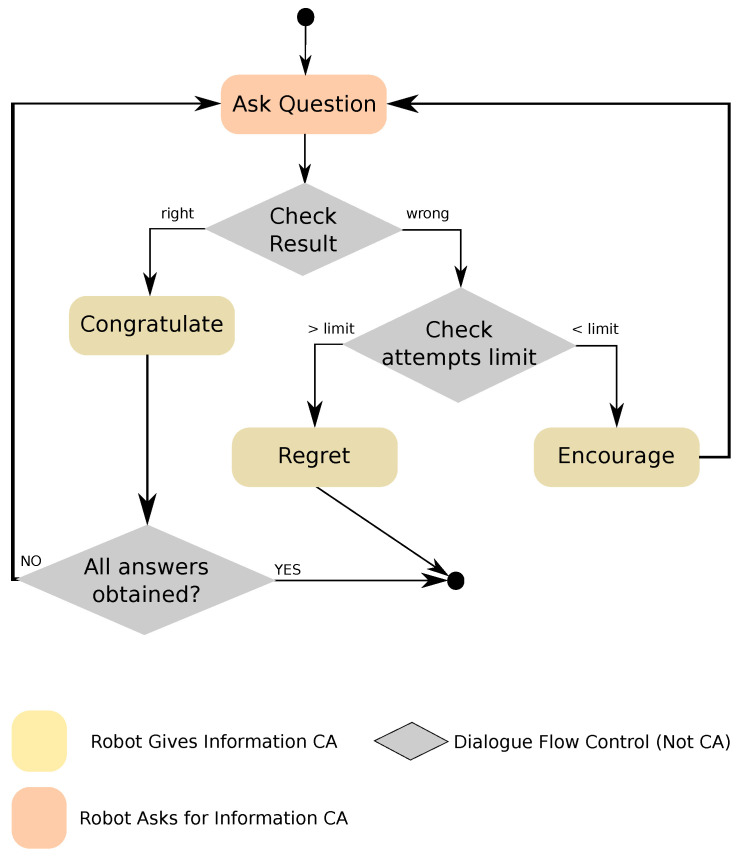
Flow diagram for the right–wrong question Complex Communicative Act (CCA). In this example, the CCA expects a sequence of answers in a given order.

This CCA, shown in Figure 3, extends the capabilities of the basic *Robot Asks For Information* CA with a series of new features. First, it gives feedback to the user depending on the type of question and the answer given, so the robot would congratulate the user if the answer is right, or encourage him/her to answer again if it was wrong. In addition, it allows to give multiple retries, if we are looking for a specific answer, or even asking for more than one answer, either in a specific order or not. The *Right–Wrong Question* CCA is used, for example, for playing question–answer games with users, where feedback is required after every answer. In order to apply it to different domains, on top of the parameters that can be used with the basic CAs, the developers can also parametrise how many attempts are given to the user for answering correctly.

#### 5.2.2. Question with Confirmation CCA

**Figure 4:**
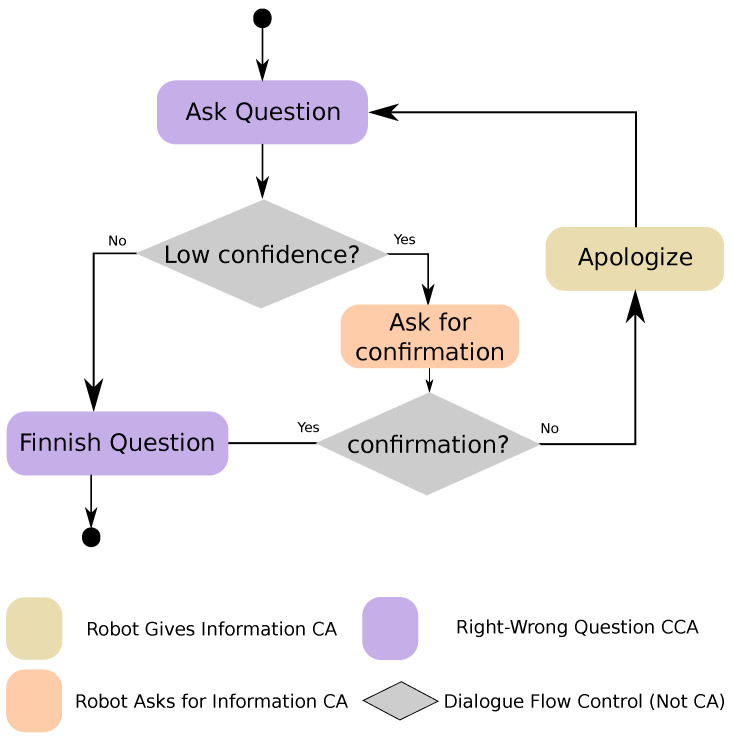
Flow diagram for the question with confirmation CCA. This CCA asks the user for an explicit confirmation of the answer if the confidence of the answer recognition is below a given threshold.

As shown in Figure 4, this CCA is designed to manage errors while processing the user’s answer to a question by asking for confirmation when the confidence is low. If the modules in charge of acquiring the user’s input cannot get a result with enough confidence, then the CCA would ask the user to confirm if the input detected is right. If the user answers affirmatively, then the *Question With Confirmation* CCA continues with the normal question process. If the answer is negative, then it utters an apology for having made a mistake and then repeats the question. The configuration parameters used are the same ones used by the *Right–Wrong Question* CCA.

#### 5.2.3. Switching Mode Question CCA

**Figure 5:**
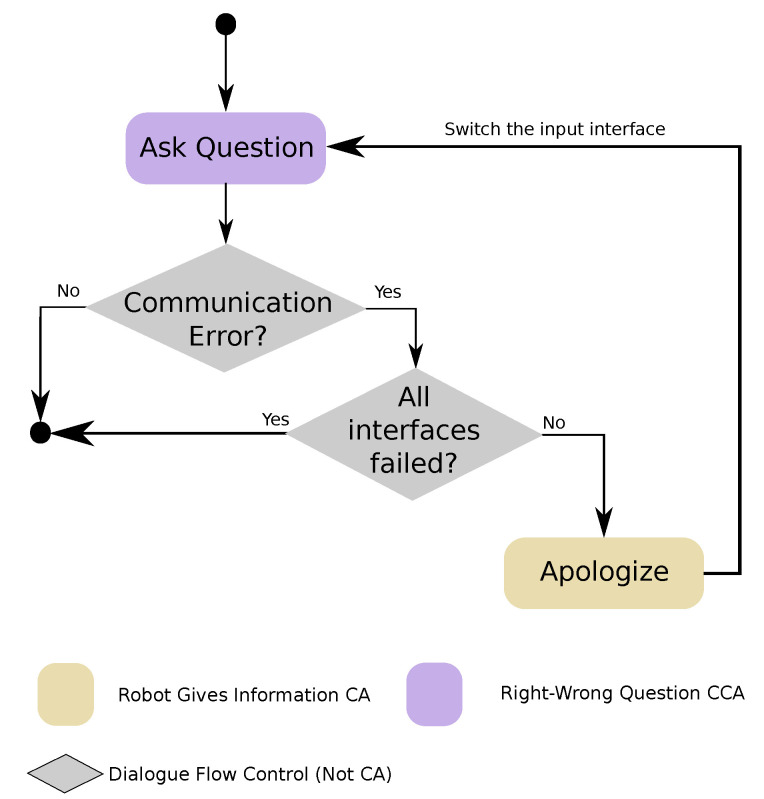
Flow diagram for the switching mode question CCA. This CCA can be configured with multiple input modes, and then it switches from mode to mode in case of communication problems.

This CCA, as shown in Figure 5, is used to ask a question and wait for the answer using one input channel; if the user cannot respond using this channel, then the CCA repeats the question and switches to the next available input channel. The order for the communication channels can be configured, or it uses a predefined order based on the available channels. If the question is answered without problems, then the result is returned. However, if a problem arises with the selected input interface (e.g., if the robot is not able to acquire an answer with enough confidence), then the CCA changes the input channel. This process continues until a satisfactory answer is obtained or until all configured channels fail to provide an answer with a confidence value high enough. In addition to the possibility of using a sequence of input methods, this CCA uses the same parameters than the *Right–Wrong Question* CCA. Notice that this CCA uses, in turn, the *Right–Wrong Question* CCA. This is a clear example of the re-usability that our approach provides.

#### 5.2.4. Manage Multimedia Content CCA

As stated before, the robot Mini is endowed with a tablet screen that can be used as an additional communicative mode, both as an output channel to show multimedia data (e.g., text, images or videos) and as an input channel to receive user commands through the touch screen. When displaying the multimedia content, the users need to be able to control that content.

**Figure 6:**
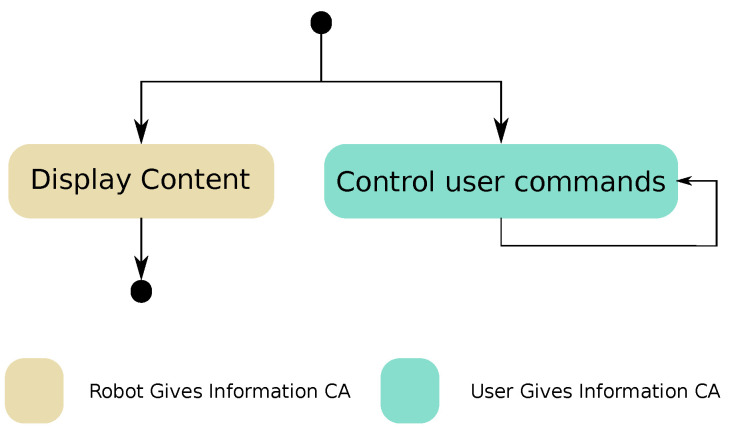
Flow diagram for the manage multimedia content CCA. This CCA sends the content to the touch screen and allows the user to control the execution of the content with voice commands.

The aim of this CCA, shown in Figure 6, is to control the multimedia content that the robot displays on the tablet. A *Robot Gives Information* CA is used to send content to the screen. At the same time, a *User Gives Information* is activated in parallel to manage all the commands that the user needs for pausing, stopping and controlling other aspects of the multimedia content. For example, we use this CCA to reproduce videos and audiobooks on Mini’s tablet, as a way of entertaining the user. The *Manage Multimedia Content* CCA can be configured using the same parameters required by the basic CAs *Robot Gives Information* and *User Gives Information*.

#### 5.2.5. Communication Warning CCA

In multimodal human–robot dialogues, robots use different communicative channels to interact with a person. Mini uses different channels through different interfaces, both inboard (e.g., voice through text-to-speech and ASR engines) and external (e.g., visual and touch channels through an external tablet). When one of these interfaces fails, the communication through the corresponding channel is affected.

**Figure 7:**
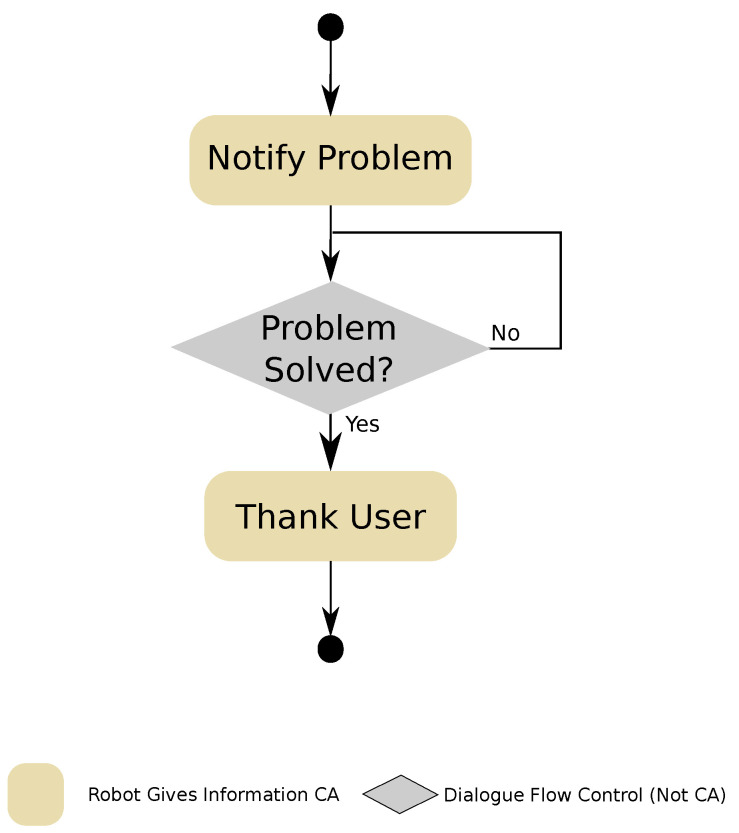
Flow diagram for the communication warning CCA. This CCA is requested if external peripherals used by the robot stop working. The CCA requests help from the user, waits for the problem to be solved and then thanks the user.

This CCA, shown in Figure 7, is designed to warn the user about a problem with one of the robot’s communication channels. First, it warns the user about the problem, gives them a set of instructions for solving the problem or instructs them to seek help, if the problem cannot be solved. At the same time, the CCA notifies the rest of the robot’s applications about the problem with the communication channel. Then it waits until the problem has been solved. Once the broken communication channel is working again, the *Communication Warning* CCA thanks the user for solving the problem. This CCA is for internal use only, therefore it is not parametrisable by the developers of applications.

## 6. Case of Use

**Figure 8:**
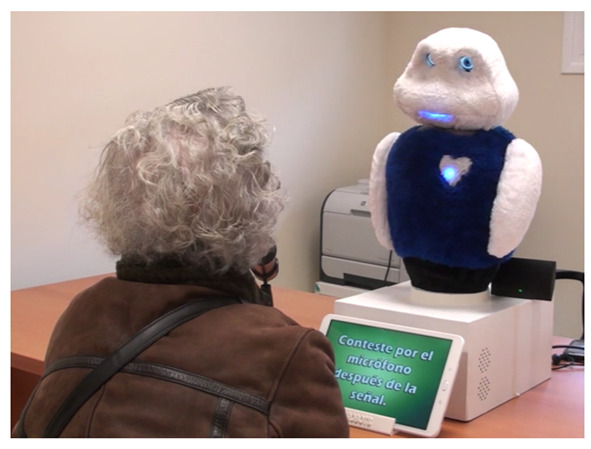
An older adult interacting with Mini.

In this section, we present a case of use with the robot Mini (presented in Section 4) interacting with four senior people. An example of this can be seen in Figure 8. This case of use demonstrates the capabilities of the proposed approach to dialogue modelling in real interactions. Moreover, we measured the response time of the CAs and the number of times that the off-the-shelf recovery mechanisms were employed. The experiments were reviewed and approved by the medical personnel of the institution in which the trials were conducted. In addition, all participants gave their signed consent before participating in the experiment.

In the case of use, the robot Mini was placed in a room in a standby state, giving the impression of being asleep. Then, a user entered the room, sat in front of Mini and woke her up by touching the robot. Mini greeted the user and asked them to select among the variety of applications that it offers. The application selected (*App${}_{exercise}$*) is designed to perform cognitive stimulation exercises from different categories (memory, perception, etc.) with the older adults. It provides a series of challenges aimed to stimulate his/her brain and slow down the cognitive deterioration.

During the execution of the cognitive stimulation exercise, a second application was active at the same time. This application, *App${}_{warnings}$*, generates personalized warnings aimed to help the user remember scheduled events. For example, the user needs to take his/her medicines at 6 p.m. According to the priorities presented in Section 4.2, the *App${}_{exercise}$* has medium priority, while the *App${}_{warnings}$* has high priority. Following, we describe how some of the most relevant features of our system are applied during this interaction with the robot Mini.

### 6.1. Interaction 1: Waking up Mini

When the user sat in front of Mini and touched it, the robot greeted them with a *Robot Gives Information* CA and then asked them what to do next, using a *Robot Asking for Information* CA. This CA was parametrised to allow the user to select the application through voice commands or through a menu displayed in the robot’s tablet. The user selected a cognitive stimulation exercise and Mini started the *App${}_{exercise}$*. The app then requested the activation of a *User Gives Information* CA for controlling the stimulation session (stopping, pausing and resuming it). The *App${}_{exercise}$* uses this particular CA configured as *continuous* to give the user the possibility of issuing any of the commands at any time during the exercises. Then, the robot explained the rules of the first exercise using a *Robot Gives Information* CA that is configured to used utterances and the motion of Mini’s body to communicate the instructions. This exercise tests the user awareness by asking them a series of questions: What season are we in?, What month are we in? What month day is today? and What week day is today?

After the instructions ended, the application started asking questions using the *Switching Mode* CCA. This CCA was configured to receive answers through voice, switching to the tablet if problems arise with the verbal communication. When the user gave the right answer, the robot used a *Robot Gives Information* CA to congratulate the user, and this CA was parametrised with a gesture that makes Mini raise the arms, display a happy look, change the colour of the heart and utter a congratulation message. Then, the *App${}_{exercise}$* moved on to the next question.

#### 6.1.1. Interaction 2: Communication Problems

**Figure 9:**
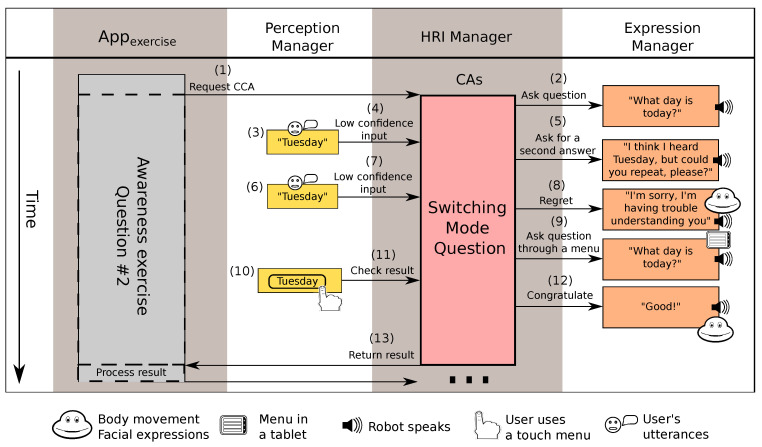
Second question of the awareness exercise. The boxes represent actions performed by the different agents and the arrows represent the connections between these actions. Numbers will be referenced in the text to help the reader.

For asking the second question in the awareness exercise, represented in Figure 9, the *App${}_{exercise}$* requests the activation of a *Switching Mode Question* CCA (see 1, Figure 9). This CCA is configured to ask the question ”What day is today?” using a robot’s utterance (see 2, Figure 9). This time, the user tried to give the right answer (see 3, Figure 9), the ASR was able to recognise the speech, but this recognition had low confidence (see 4, Figure 9). Then, the robot told the user what the ASR had recognised but asked them to repeat it anyway (see 5, Figure 9), just to be sure. The user kept trying to give the right answer (see 6, Figure 9) but the robot was unable to understand them (see 7, Figure 9), so the CCA switched the interaction mode and repeated the same question, only this time asking the user to answer through a menu displayed in a touch screen (see 8 and 9, Figure 9). The change of interface made the communication problems disappear, so the user was able to finally give the correct answer (see 10 and 11, Figure 9). Finally, the *Switching Mode Question* CCA congratulated the user (see 12, Figure 9) informed the application of the success (see 13, Figure 9).

#### 6.1.2. Interaction 3: Failing to Answer in Time

**Figure 10:**
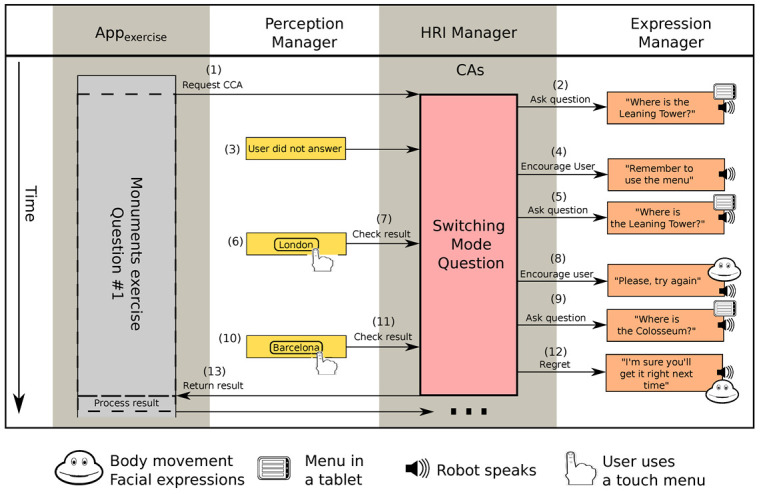
First question of the monuments exercise. The boxes represent actions performed by the different agents and the arrows represent the connections between these actions. Numbers will be referenced in the text to help the reader.

Another exercise, which was designed for testing the user’s executive function, consisted on showing the user a series of pictures of monuments and asked them in which city were they located. The user had to answer through a menu displayed in the tablet. In the first question, shown in Figure 10, the exercise used a *Switching Mode Question* CCA (see 1, Figure 10) to ask about the location of the Leaning Tower (see 2, Figure 10), which is located in the Italian city of Pisa. The user failed to answer in time (see 3, Figure 10). Then, the *Switching Mode Question* CCA encouraged them to answer, and reminded them to answer using the menu in the tablet (see 4, Figure 10). The robot repeated the question (see 5, Figure 10), but the user was not sure of the right answer, so gave a wrong answer: *London* (see 6, Figure 10). As a result that the answer was wrong (see 7, Figure 10), the *Switching Mode Question* CCA encouraged them to try again (see 8, Figure 10) and then repeated the question (see 9, Figure 10). The user then answered: *Barcelona* (see 10, Figure 10). The answer was wrong again (see 11, Figure 10) and Mini tried to cheer the user with an encouraging message and by changing the expression of the eyes and the colour of the heart (see 12, Figure 10). Finally, the CCA informed the *App${}_{exercise}$* that the user gave a wrong answer (see 13, Figure 10). The application them moved on to the next question.

#### 6.1.3. Interaction 4: Pausing CA with Lower Priority

**Figure 11:**
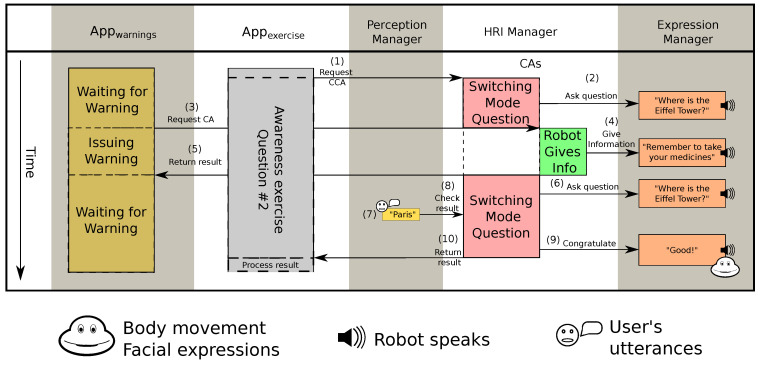
Example of a situation with several CAs with different priorities. The boxes represent actions performed by the different agents and the arrows represent the connections between these actions. Numbers will be referenced in the text to help the reader.

The next interesting interaction is presented in Figure 11. During a question in the monuments exercise, which asked the user about the location of the Eiffel Tower (see 1 and 2, Figure 11), the *App${}_{warnings}$* detected it was already 6 p.m. and requested the HRI Manager a *Robot Gives Information* CA with high priority to remember the user that it was time for taking their medication (see 3, Figure 11). As a result that the *Switching Mode Question* CCA requested by the *App${}_{exercise}$* had medium priority, the HRI Manager stopped it, stored it and then executed the new CA. After, the robot issued the warning about their medication (see 5, Figure 11), the HRI Manager extracted the prior active CA stored in the priority queue, and restarted it continuing with the exercise. The robot repeated the same question (see 6, Figure 11), the user gave the right answer (see 7 and 8, Figure 11) and the *Switching Mode Question* CCA informed the *App${}_{exercise}$* that the answer was correct (see 10, Figure 11). The robot repeated the congratulation presented in the first question of the awareness exercise (see 9, Figure 11).

#### 6.1.4. Interaction 5: Recovering from a Network Error

**Figure 12:**
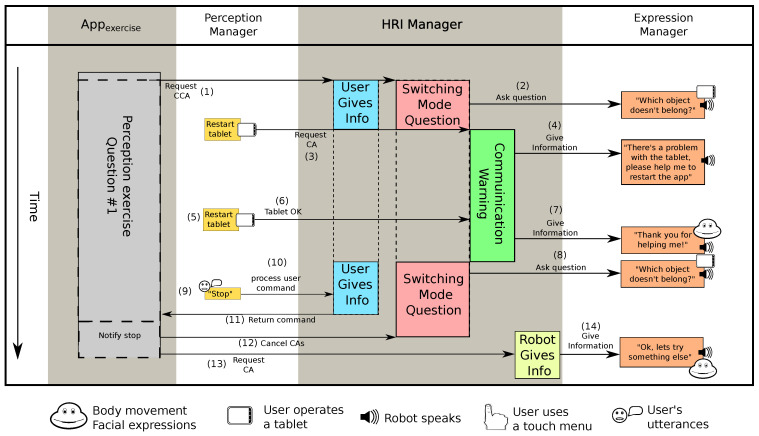
Fourth question of the exercise. The boxes represent actions performed by the different agents and the arrows represent the connections between these actions. Numbers will be referenced in the text to help the reader.

In this final notable interaction, the exercise presented to the user had the goal of testing his/her perception. A series of objects were presented in the tablet and the user had to select the one that was not related to the others. For each question, this exercise activates two CAs (see 1, Figure 12): (i) a *User Gives Information* CA, that allows the user to suspend the exercise at any time, and (ii) a *Switching Mode Question* CCA. In the first question, the exercise showed a blue car, a blue pencil and an orange ball, and asked what object did not belong (see 2, Figure 12). The answer was the ball, as it was the only object that was not blue but, during this question, due to a problem with the network, the connection between the robot and the touch screen was broken.

The tablet publishes a signal at a certain rate so the robot can control if it is working properly. When the Perception Manager stopped receiving this signal, it requested a *Communication Warning* CCA to inform the user about the problem (see 3, Figure 12) (The HRI Manager monitors all the input interfaces in the robot and informs when a communication channel cannot be used.). The HRI Manager, then, stopped the execution of the current question, stored the *Switching Mode Question* CCA and activated the *Communication Warning* CCA that notified the user about the problem with the tablet (see 4, Figure 12). The CCA asked the user to restart the app running in the tablet and then waited. Once the user restarted the app (see 5, Figure 12) and the HRI Manager received confirmation that it was working again (see 6, Figure 12), the CCA concluded using a *Robot Gives Information* to thank the user for their help with a happy face and an utterance (see 7, Figure 12).

At this point, the HRI Manager resumed the execution of the *Switching Mode Question* CCA. The question was asked again (see 8, Figure 12) but because the user was already tired, she decided to take the initiative and asked the robot to stop the session (see 9, Figure 12). The *User Gives Information* CA received this request (see 10, Figure 12) and notified the *App${}_{exercise}$* application (see 11, Figure 12), which requested the HRI Manager to cancel all active CAs related to the session (see 12, Figure 12), and confirmed the user that the exercise was over through a *Robot Gives Information* CA (see 13 and 14, Figure 12). With this, the execution of the app ended.

### 6.2. Response Time

As previously mentioned, DSs in social robots are aimed at providing a human–robot interaction that is perceived as natural by the human peer. In this regard, the response time of the system is crucial. In human–computer interaction, some studies reported that the preferred response time is less than one second [34,35]. Other researchers established the “two second” rule as a guideline for designing system response times [36]. In 2009, Shiwa et al. demonstrated that this rule can be applied to the field of Human–Robot Interaction [37]. With all of this in mind, we can confidently set a threshold of one second as an acceptable response time for our system.

To validate our system, we have measured the response time of the different CAs used in the case of use. The system automatically registers the time for every step of the robot’s interactions. We used these registers to measure the response time of the CAs. We considered three different situations that affect the perception of the interaction by the user:

- The response time for processing the activation request. In this case, the response time considers the time between the HRI Manager receives the request and the management of the corresponding interfaces. This management can be either the configuration of the needed input interfaces (for example, in a *User Gives Information* CA, the display of a menu in the touch screen), or the beginning of the execution of a robot’s expression (for instance, in a *Robot Gives Information* CA, the execution of the congratulation gesture). In certain situations, this response time can include the time required to disrupt other CAs with lower priority. This response time is present in all CAs.
- The response time for processing the user’s input messages. In this case, depending on the CA and its parametrization, the response time is measured from the moment Mini receives the message (e.g., the user touches the robot or the robot perceives a user’s utterance), to the moment the CA generates a response expression. This time exists only in the CAs that expect an input from the user.
- The response time for completing the CA. Once the execution of the CA is completed, it sends the result of the interaction to the module that requested its activation. The time between the end of the interaction and the communication of the result is the response time for completing the CA. This response time is present in all CAs.

It is important to notice that the response time can vary significantly between CAs because some of them (for example, *Switching Mode Question* CCAs) are more intricate than others. Even for the same CA, the response time can vary from one execution to other due to other CAs being executed in parallel with different priorities.

**Figure 13:**
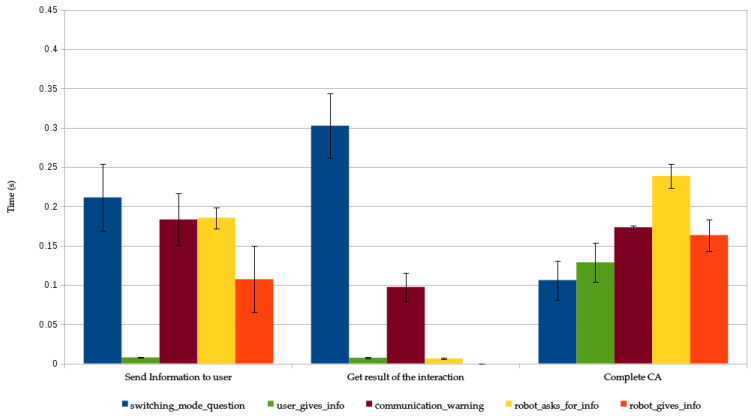
Evaluation of the CAs’ response time. Bars represent the mean value in seconds and whiskers represent standard deviation.

The results are presented in Figure 13 where bars represent the mean value of the response time and error bars are the standard deviation. We can observe that, in all cases, the response time is below one second (which is the threshold that we set based on the works evaluated at the beginning of this section), so we can fairly say that our system fulfil with the response time requirement.

### 6.3. Use of the Recovery Mechanisms

The interactions with the users were taped and we analysed the videos to extract global statistics about the quality of the interaction. In total, the seniors interacted with Mini for 71 mins. (the combined length of all the videos) and 354 CAs, both basic and complex, were used by the high-level modules of the robot. A total of 88.42% of the interactions were completed without problems (313 of the 354 CAs), while the remaining 11.58% required the use of at least one of the built-in recovery mechanisms to solve communication problems (41 of the 354 CAs). Table 3 summarises all the information presented here.

The CAs resorted to one of the different recovery mechanisms in 65 occasions (some CAs had to handle more than one of the considered unexpected situations). In 29 occasions (44.62% of all the recovery mechanisms usage), the interaction through voice was unsuccessful due to the inability of the ASR for providing an acceptable recognition (the ASR returned recognition errors in 17 occasions and low confidence value in the other 12 times). The CAs had to encourage the users to provide an answer 19 times due to the microphone was unable to capture the user’s answer, the user did not know the answer, or he/she took too much time to respond (this represents the 29.23% of all the situations managed by the recovery mechanisms). Finally, some of the responses given by the users were not the answers that the system was expecting, so the CAs had to ask the users for a new answer in 17 situations, which represents a 26.15% of all situations. The usage of the different recovery mechanisms is shown in Table 4.

### 6.4. Limitations of the System

Although our approach has proven capable of managing one-to-one interactions successfully, as well as being able to overcome unexpected problems, it also presents some limitations. The HRI Manager relays in the applications to design the logic of the dialogues. This can lead to rigid or repetitive interactions, depending on the interaction design followed by the roboticists. In addition, we have focused exclusively in one-to-one interactions, as those are the scenarios for which our robotic platforms are designed. As a result of this, our approach is not able to manage interactions in which multiple users are present. Finally, despite this paper is not focused on the Perception Manager, this module provides the aggregated information that the HRI Manager processes in the different active CAs. Currently, multimodal fusion is not implemented yet and this limits the interaction capabilities of our system. We expect that, once multimodal fusion is available, the robot will be able to conduct even more natural interactions. In addition, the use of a grammar-based ASR reduces the complexity of verbal communication, as the user verbal inputs are limited by these grammars. This could result in situations like the user giving a synonym of the answer expected by the CA, but being told by the robot that it could not understand him/her because that particular synonym is not contemplated in the grammar.

Regarding the results presented in Section 6, there are two limitations that have to be mentioned. First, we extracted results from interactions with only 4 users. Second, the interactions presented are from one type of application. In order to generalise the results, a more extensive evaluation with higher number of participants and more heterogeneous scenarios is needed. Despite the limitations presented in this subsection, the CA-based dialogue design approach has proven that is a valid solution for designing interactions in different domains and managing unexpected situations that arise in the dialogue, simplifying the development of applications for social robots.

## 7. Conclusions

In this work, we present an approach to human–robot dialogue modelling based on atomic interaction units called CAs. These interaction units handle small portions of the interactions and can be combined to create more complex dialogues. The basic CAs in our system can manage conversations in which either the robot or the user have the initiative and allow for either giving information to the peer without the initiative or asking for information that the speaker with the initiative needs. The CAs also offer some built-in recovery mechanisms that manage frequent problems that might arise during any interaction (user disengagement and communication errors). We also present complex CAs, which are a series of structures that appear in multiple dialogues, and that are built as a combination of CAs. The main advantage of these CCAs is their re-usability, further simplifying the development of new dialogues. Both CAs and CCAs can be parametrised to meet the requirements of different scenarios. Using this approach, the design and development of new human–robot interactions can be done easier.

In our architecture, CAs take care of all the aspects of interaction that do not require task-related information which simplifies the creation of new dialogues. The HRI Manager is the module in charge of activating and deactivating CAs by request of the applications and of controlling that there are no conflicts among all the active CAs. Our system controls changes on dialogue topic and initiative by allowing to execute multiple CAs and CCAs in parallel.

The most important contributions of this paper are: (i) The development of a two-level multimodal dialogue management strategy based on atomic interaction units called Communicative Acts, or CAs; (ii) the implementation of the HRI Manager, the module in charge of managing the parametrisation and execution of the CAs, as well as solving certain communication problems that might arise during the interactions (user not responding, answer given by the user not matching the answer expected or malfunctions of the robot’s output interfaces, among others); (iii) The development of a library of complex CAs, or CCAs that can be reused and parametrised in order to adapt them to different situations. These CCAs are combinations of basic CAs, and prove the modularity and re-usability of the approach presented.

The system presented in this paper has been implemented in a robotic platform that is being developed for commercialization purposes. As a result of this, all the source code is part of a proprietary closed-sourced software package and it is developed under a privative licence.

To show how the proposed approach works, we have presented a case of use where real users interact with a social robot designed for assisting older adults with a certain degree of cognitive impairment. During the interactions, the users were able to communicate without any problem in 88% of the cases, while the system was able to solve the problems that arose in the other 12% of the situations successfully. In this case, the CAs used in the dialogues have been configured following the guidance of the subject matter experts (caregivers, physician and relatives). It is important to notice that, in different scenarios, these parameters can be easily configured to meet the particular requirements thus, this approach can be applied easily to other scenarios where one person interacts with a robot.

Moreover, all CAs used in the case of use kept their response time below one second and thus it guarantees that our system fulfils the two second rule. That proves that our solution is able to handle multimodal human–robot interactions fast enough to provide interactions that feel natural to the user.

## Figures and Tables

**Table 1:** Comparison among the works presented in Section 2. Each approach has been evaluated according to scalability, multi-modality and flexibility.

Approach	Ref.	Scalability and Re-Usability	Multi-Modality	Flexibility
State-based	[16]	Combine and parametrise the interaction patterns adequately. This patterns can be reused among tasks	Yes	Interaction adapted through parametrisation of the patterns
Frame-based	[10,17]	Describe the new task with the description language of choice. The slot-filling techniques are reused among tasks	No ([17]), Yes ([10])	Predefined responses
	[18]	Build new domain ontologies, reuse the ontology-based rules for dialogue management	No	Responses are defined in the ontologies
Plan-based	[14]	Design the domain-dependent aspects, using dialogue agents, while reusing the domain-independent aspects	No	Responses are implemented in the dialogue agents
Probabilistic-based	[9]	Build and parametrise new probability rules. Some rules can be reused for different domains	Yes	Responses can be adapted through the probabilistic rules
	[19]	Build new domain ontologies. Generic aspects of the dialogue are reusable	No	System responses are generated through NLG
	[20]	Increase the database. Statement response module can be reused among domains	No	Predefined responses
Agent-based	[21]	New datasets and task agents would be required. This approach require smaller datasets than usual. The example manager module is common to all tasks	No	Uses template-based NLG
End-to-End	[22,23,24,25,26,27,28,29]	Develop new datasets and agents (if applied). The model is common to all tasks. Some agents may be generic	No	Use NLG for generating the system response (template-based or model-based)

**Table 2:** Basic Communicative Acts (CAs) that have been currently developed.

		Initiative	
		Robot	User
**Intention**	**Giving Information**	*Robot Gives Information*	*User Gives Information*
	**Requesting Information**	*Robot Asks for Information*	*User Asks for Information*

**Table 3:** Global statistics about the interactions.

Duration of the Interactions	71 min	
Successful interactions	313	88.42%
CAs requiring recovery mechanisms	41	11.58%
**Total**	354	100%

**Table 4:** Usage of the recovery mechanisms.

ASR failures	17	26.15%
Answer recognitions with low confidence	12	18.46%
User not responding	19	29.24%
Wrong answer provided by the user	17	26.15%
**Total**	**65**	100%
